# Development of a Graphene-Based Surface Plasmon Resonance Optical Sensor Chip for Potential Biomedical Application

**DOI:** 10.3390/ma12121928

**Published:** 2019-06-14

**Authors:** Nur Alia Sheh Omar, Yap Wing Fen, Silvan Saleviter, Wan Mohd Ebtisyam Mustaqim Mohd Daniyal, Nur Ain Asyiqin Anas, Nur Syahira Md Ramdzan, Mohammad Danial Aizad Roshidi

**Affiliations:** 1 Institute of Advanced Technology, Universiti Putra Malaysia, UPM Serdang, 43400 Selangor, Malaysia; nuralia.upm@gmail.com (N.A.S.O.); silvansaleviter94@gmail.com (S.S.); wanmdsyam@gmail.com (W.M.E.M.M.D.); nurainasyiqinanas@gmail.com (N.A.A.A.); roshididanial@gmail.com (M.D.A.R.); 2 Faculty of Science, Universiti Putra Malaysia, UPM Serdang, 43400 Selangor, Malaysia; nursyahira.upm@gmail.com

**Keywords:** graphene-based material, quantum dots, surface plasmon resonance, biosensor

## Abstract

The emergence of unintentional poisoning and uncontrolled vector diseases have contributed to sensor technologies development, leading to the more effective detection of diseases. In this study, we present the combination of graphene-based material with surface plasmon resonance technique. Two different graphene-based material sensor chips were prepared for rapid and quantitative detection of dengue virus (DENV) and cobalt ion (Co^2+^) as an example of typical metal ions. As the fundamental concept of surface plasmon resonance (SPR) sensor that relies on the refractive index of the sensor chip surface, this research focused on the SPR signal when the DENV and Co^2+^ interact with the graphene-based material sensor chip. The results demonstrated that the proposed sensor-based graphene layer was able to detect DENV and Co^2+^ as low as 0.1 pM and 0.1 ppm respectively. Further details in the detection and quantification of analyte were also discussed in terms of sensitivity, affinity, and selectivity of the sensor.

## 1. Introduction

In this era of ever-increasing populations, the demand for the improvement of living standards may cause some serious environmental issues, e.g., chemical exposure, electromagnetic radiation, and air and water pollutions [1,2], and, thus, may lead to some high-risk health problems such as chronic disease and metal poisoning [3,4,5]. Therefore, a lot of clean earth campaigns, and occupational health and safety programs have been initiated by the government to control diseases, food production, atmospheric pollution, and water safety [6,7,8,9,10]. One of the most common deadliest diseases that can be found throughout the world is the mosquito-borne disease known as the dengue virus (DENV), which is transmitted by female *Aedes* mosquito, *Aedes aegypti*. Although recent laboratory techniques have contributed to treating the disease, the efficient and rapid detection for DENV is still of great significance to detect the dengue virus within the viremia and early febrile phase of infection [11,12,13,14,15,16,17]. The above-mentioned laboratory techniques, such as enzyme-linked immunosorbent assay (ELISA), reverse transcriptase-polymerase chain reaction (RT-PCR), and real-time polymerase chain reaction (qPCR), have some issues in terms of time-consuming, trained workers, complex preparation, and high-cost reagents [18,19,20,21,22,23,24].

The next important health problem to address is metal toxicity, which can be found in canned drinks and foods [25,26], vehicle emissions [27], cosmetics [28,29] and industrial waste [30,31]. The excessive heavy metal ions released by industries into the environment causes pollution in aquatic and terrestrial systems, such as forests, rivers, lakes, seas, and soils that consequently bring an effect to natural cycles [32]. Despite their overall scarcity, some heavy metal ions, such as iron, copper, zinc, and cobalt, are known to be nutritionally essential to the human being. However, the excessive level of essential free metal ions can cause poisoning and potentially lead to serious health problems. Other heavy metal ions, such as mercury, lead, and cadmium, are no exception and are dangerous and poisonous even in low consumptions [33,34]. Due to that, many conventional techniques, such as electrochemical microfluidics, fluorescence sensor, colorimeter sensor, and enzyme-based biosensor, for metal ions detection have successfully reduced the detection limit for cadmium, mercury, and cobalt to very low concentrations of 11 ppb, 3 ppb, and 2 ppm, respectively [35,36,37,38,39]. Although these methods have adequate sensitivity, they involve long-time measurement, complex preparation, and expensive instrumentation. 

A rising surface-sensitive technique, surface plasmon resonance (SPR)-based biosensors, has drawn much attention from many researchers due to its ease of preparation, real-time detection, high sensitivity, fast measurements, and label-free method [40,41,42,43,44]. This technique is especially suitable for in situ studies of molecular interactions and binding specificity. Owing to that, SPR-based biosensors have come out as a high-potential sensor in medical diagnostics and environmental monitoring [45,46,47,48,49,50]. A key parameter in producing the most sensitive SPR-based biosensor is by coupling SPR with the novel biomolecules immobilized on the gold thin film. Another equally important parameter that governs the sensitivity of the SPR sensor is the transport of low concentration target molecules to the sensor surface. Several methods have been employed by other researchers to improve the sensitivity of biosensor [51,52,53]. According to the theoretical phenomenon of SPR, the sensitivity of the SPR sensor is governed by binding between the analyte and the surface binding interactions at the gold surface. The binding between them causes the changes in the refractive index on the gold surface, and, thus, would able to excite the surface plasmon wave to produce the SPR signal [54,55,56]. Nowadays, graphene and its derivatives, i.e., graphene and reduced graphene oxide, have generated tremendous interest because of their biocompatibilities, large surface area, non-toxicity, enriched functional groups, and excellent optical, electrical, and mechanical properties [57,58,59,60,61]. In this sense, graphene-based materials have emerged as potential sensing platforms for the immobilization of biomolecules or for the detection of various analytes [62,63,64,65,66,67]. Since then, recent works on graphene-SPR-based materials have been applied in many fields. Their important sensing parameters, such as sensitivity, detection limit, and binding affinity, are discussed and summarized in Table 1. 

In view of biological sensing, Wang et al. (2011) have developed the graphene-based SPR sensor with the aptamer binding to monitor alpha-thrombin in the detection chamber [68]. The novel concept of graphene was further utilized for the detection of Tuberculosis bacilli in 2012. Chiu et al. demonstrated a novel Au-self assembled monolayer-graphene nanocomposite, yielded a highly sensitive analysis when compared to the conventional Au/Cr-based SPR chips [69]. In a publication by Aksimsek and Sun, the sensitivity of graphene-molybdenum disulfideconjugated with ssDNA was analyzed with a silver substrate [70]. They found that the shift in the resonance angle and sensitivity were significantly improved by 13% with the number of graphene layers. Other work on the detection of *Mycobacterium tuberculosis* DNA (deoxyribonucleic acid) hybridization in SPR sensor was introduced by Prabowo et al. (2016), and yielding the best detection limit of 28 fM [71]. In 2017, Jiang et al. used goat anti-rabbit IgG-modified reduced graphene oxide to detect rabbit IgG. The lowest detection limit of 0.0625 µg/mL rabbit IgG was successfully obtained, compared to the commercial SPR apparatus of 0.3125 µg/mL [72]. Next, the carboxyl-functionalized graphene oxide composite was developed for the detection of protein bovine serum albumin. Experimental results revealed a strong linear graph of R^2^ = 0.973 with a detection limit of 0.01 pg/mL [73]. In 2017, Jamil et al. reported on the MoS2/graphene-based SPR sensor for urea detection. The results showed that the sensitivity of the SPR sensor was greatly enhanced by adding the number of MoS2 and graphene layer [74]. The first graphene-based SPR sensor for galection-3 detection was reported by Primo and co-workers in 2018. The preparation of the sensor film began by self-assembling the gold surface with four bilayers of poly(diallyldimethylammonium chloride) and graphene oxide, followed by the covalent attachment of 3-aminephenylboronic acid (3ABA). It was found that the detection limit of the proposed sensor was 2.0 ng/mL [75]. Today, the combination of optical fiber and SPR sensor has been capturing increasing research interest. Gong et al. have developed a D-shaped plastic optical fiber surface plasmon resonance based on the graphene for the detection of glucose and DNA. The obtained proposed sensor’s sensitivity was 1227 nm/RIU (refractive index unit) for glucose detection. Meanwhile, for DNA detection, a satisfactory linear response with an R^2^ of 0.996 was achieved in the concentration range of 0.1 nM to 1 µM [76]. 

As can be noticed from Table 1, no research for DENV detection using graphene-based material incorporated with an SPR sensor has yet been reported. It is desirable that the graphene-SPR-based materials technique has a high potential in biosensing, thus, for the first time, we developed a novel graphene-based composite material to detect DENV quantitatively using the SPR technique. The SPR sensing platform of gold/cadmium sulfide quantum dots-reduced graphene oxide (Au/CdSQDs-rGO) film was expected to improve their sensitivity and limit of detection, thus be well adapted to be used for quantitative clinical analysis. 

Meanwhile, in the first work on heavy metal ion detection, Lokman et al. (2014) successfully detected Pb^2+^ using Au-chitosan-graphene oxide (Au/CS/GO)-based SPR sensor. They compared the performance of the developed SPR sensor with and without graphene oxide (GO). Even though both thin films can detect Pb^2+^ as low as 0.03 ppm, a rougher surface was observed when GO was added to Au/CS, which was believed it could improve the adsorption of Pb^2+^. As a result, Au/CS/GO showed wider Pb^2+^ range with higher sensitivity [77]. Furthermore, a work by Nawi et al. used gold nanoparticles decorated graphene oxide-polyaniline nanocomposite (AuNPs/GO/PANI) deposited onto indium-tin-oxide glass. The nanocomposites have high sensitivity towards Pb^2+^ detection when exposed to different concentrations of Pb^2+,^ ranging from 0.03 ppm to 3 ppm [78]. Another similar work by Kamaruddin et al. implemented multiple metallic layers Au-Ag-Au to the CS-GO layer, which was also used to detect Pb^2+^, and there was a shift in SPR angle increased up to 3.5° compared to the Au-CS-GO layer only. A high sensitivity value of 2.05° ppm^−1^ using the SPR sensor was obtained [79]. Once again, Kamaruddin and his co-workers used the same active layer, Au/Ag/Au/CS-GO incorporated with SPR to detect Hg^2+^. They observed higher sensitivity of the previously developed sensor towards Pb^2+^ compared to Hg^2+^, thus, they calculated the binding affinity and found that CS-GO was more favorable to Pb^2+^ [80]. Besides, a work for K^+^ detection using SPR sensor was reported by Zainudin et al. (2018) where valinomycin-doped chitosan-GO (C-GO-V) was used as an active layer. A preliminary test with Au thin film for K^+^ detection showed no shift in the resonance angle. However, when replaced with Au/C-GO-V, they successfully yielded the lowest detection limit of 0.001 ppm with a sensitivity value of about 0.00948° ppm^−1^ [81]. Daniyal et al. used nanocrystalline cellulose modified with hexadecyltrimethylammonium bromide and GO composite (CTA-NCC/GO) as an SPR active layer to detect Cu^2+^. A high sensitivity value of 3.271° ppm^−1^ was obtained. It was found that the sensor could detect Cu^2+^ as low as 0.01 ppm [82]. Another work by Daniyal et al. used the same active layer (CTA-NCC/GO) but this time for Ni^2+^ detection. Experimental results showed that CTA-NCC/GO can detect Ni^2+^ as low as 0.01 ppm with a calculated sensitivity value of 1.509° ppm^−1^ [83]. As can be seen, all the above mentioned studies used GO-based material as an active layer. Differently, a work by Ramdzan et al. used coated chitosan/carboxyl-functionalized graphene quantum dots on top of gold (Au/Cs/CGQDs) thin film and incorporated this with SPR to detect Hg^2+^. The resonance angle was directly increased with Hg^2+^ concentrations. The Au/Cs/CGQDs can detect Hg^2+^ as low as 0.5 ppm with a sensitivity of 0.00062° ppm^−1^ [84]. Table 2 summarizes and compares graphene-SPR-based materials in metal ion sensing.

Briefly, the studies on graphene-SPR-based material for heavy metal ion detection remains inchoate, thus, further work on heavy metal ions detection, using other graphene composite material as an active layer in SPR, should be conducted since graphenes have the potential to enhance SPR performance. In the present work, chitosan-graphene oxide-cadmium sulfide quantum dots (Chitosan-GO-CdS QDs) composite thin film was developed as a sensor layer for cobalt ion detection via the surface plasmon resonance sensor. The objective of this work was to improve the detection limit of the metal ion and the sensitivity of the detection. 

## 2. SPR-Based Kretschmann Configuration 

### 2.1. Theory

**Figure 1:**
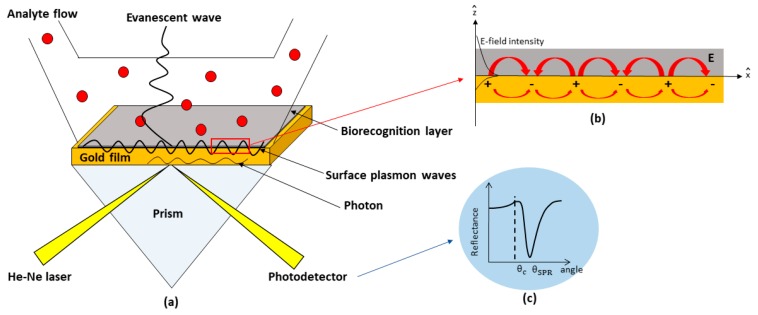
Schematic diagram of the detection-based SPR technique. (**a**) Surface plasmon waves; (**b**) Electric field components; (**c**) SPR resonance angle.

The optical phenomenon of SPR occurs when the incident He-Ne laser light is reflected from the metal-dielectric interface under certain resonance conditions. Basically, when an electromagnetic wave of incident light hits a metal film, the free electrons on the metal collectively oscillate in waves, thus producing charge density waves propagating along the metal-dielectric interface (Figure 1a). These charge fluctuations, called surface plasmon waves (SPW), are accompanied by an evanescent field and decay exponentially with distance from the surface. The waves that correspond to the evanescent field are evanescent waves and are given by [85]:

(1) $$\vec{E}=E_{o}(\hat{x}+i\hat{z})e^{i(k_{t}x\sin\theta .\frac{n_{i}}{n_{t}}-wt)}e^{-\beta \left|z\right|},$$

where $\beta =k_{t}{\left(\sin^{2}\theta .\frac{n_{i}^{2}}{n_{t}^{2}}-1\right)}^{\frac{1}{2}}$, $k_{t}$ is the wave vector in the transmitted medium, θ is the incident angle, and $n_{i}$ and $n_{t}$ are the refractive indices of the prism and transmitted medium, respectively. These waves are strongly sensitive to any refraction index alteration in the dielectric medium adjacent to the metal surface [23,86]. In order to satisfy the SPR resonance condition, the incident light must be in transverse magnetic (TM) mode, known as p-polarized light, to excite SPW. P-polarized is needed because its electric field vector is oriented perpendicular to the metal film, which can be expressed as follows:

(2) $$\vec{E}=E_{o}(\hat{x}+i\hat{z})e^{i(kx-\omega t)}e^{-k\left|z\right|},$$

where $E_{o}$, k, and ω are the amplitude, the wave vector, and the angular optical frequency of the electrical field, respectively, and $\hat{x}$ and $\hat{z}$ are unit vectors, as shown in Figure 1b. The second condition for the surface plasmon wave excitation is given by *k_x_* = *k_sp_,* where *k_x_* is $\frac{2\pi }{\lambda }n_{p}\sin\theta$, *k_sp_* is approximated as $\frac{2\pi }{\lambda }\sqrt{\frac{\varepsilon_{1}\varepsilon_{2}}{\varepsilon_{1}+\varepsilon_{2}}}$, *n**_p_*** is the refractive index of the prism, $\varepsilon_{1}^{}$ is the real part of the dielectric constant of the metal, and *Ɛ*_2_ is the dielectric constant for the dielectric medium in contact with the metal surface. The coupling of two waves vector of the evanescent wave, *k_x_*, with that of the surface plasmons, *k_sp_*, results in a sharp dip of SPR signal at resonance angle (*θ_SPR_*) as shown in Figure 1c. 

### 2.2. Experimental

**Figure 2:**
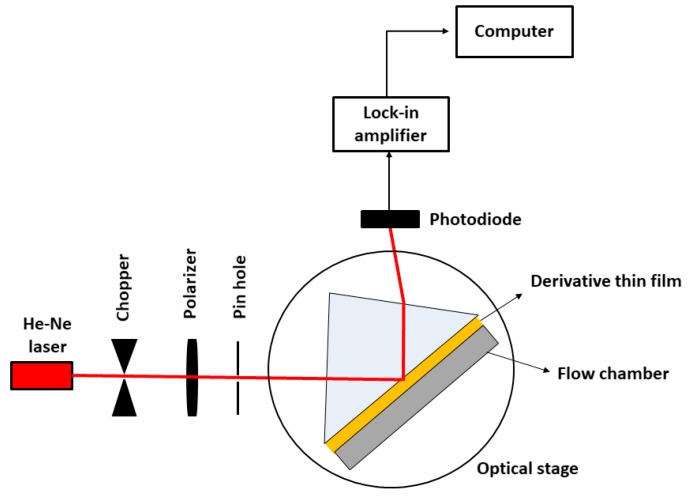
Illustration of the SPR setup system.

Figure 2 shows the SPR setup system, where a derivative thin film was attached to a prism surface using a refractive-index-matching fluid. This prism was then placed on an optical stage driven by a stepper motor from 48° to 60° (Newport MM 3000). Light from the laser source (632.8 nm, 5 mW) was then shone onto the sensor film surface. The reflected light was collected by a photodiode and then processed by a lock-in-amplifier (SR 530). A 100 µL flow chamber was attached to the derivative thin film to be filled up by the sample solution for the detection system. For the detection system, 0.1 pM DENV solution was injected into the chamber using a syringe pump. The SPR measurement was carried out continuously from 0 to 20 min for each concentration of DENV solution. 

## 3. Results and Discussions 

### 3.1. Graphene-SPR Based Materials for DENV Detection

**Figure 3:**
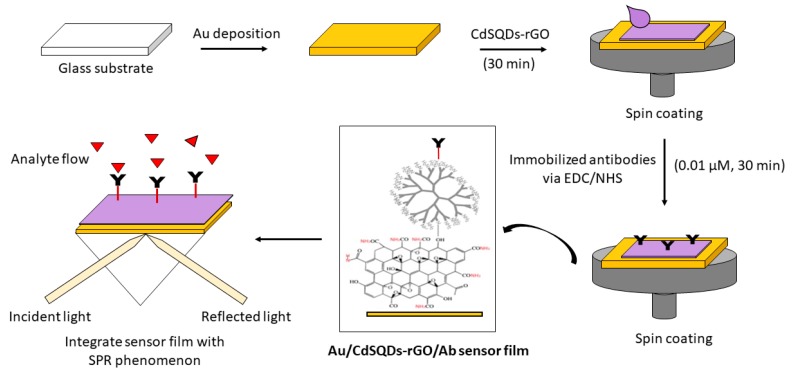
Schematic diagram of the experimental procedure.

**Figure 4:**
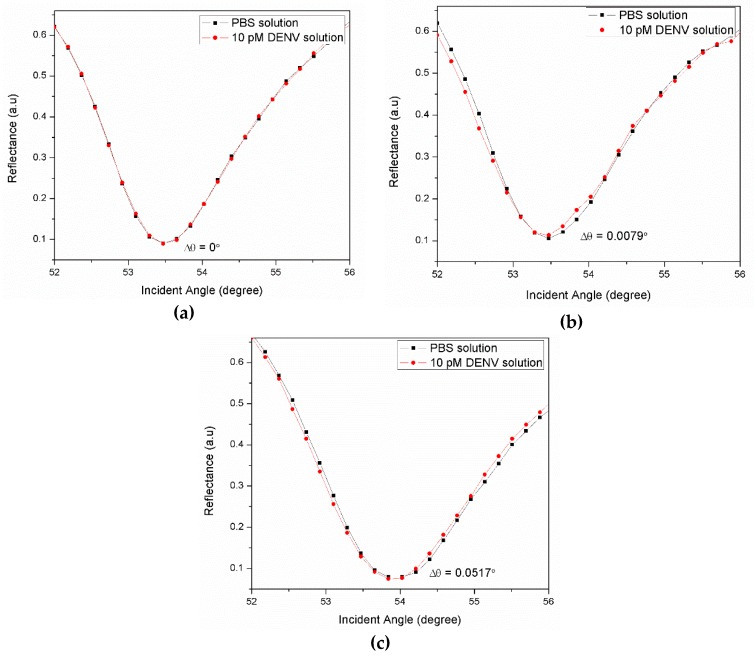
Optical reflectance for 10 pM dengue virus (DENV) solution in contact with (**a**) Au, (**b**) Au/Ab, and (**c**) Au/CdSQDs-rGO/Ab sensor film.

As illustrated in Figure 3, the first step to develop a sensor film made of gold/cadmium sulfide quantum dots-reduced graphene oxide/antibodies (Au/CdSQDs-rGO/Ab) film is to deposit a glass cover-slip (24 mm × 24 mm × 0.1 mm, Menzel-Glaser, Germany) with an Au layer using a sputter coater (50 nm, 20 mA). Next, the spin coating technique was applied to deposit the CdSQDs-rGO composite solution on top of the Au surface (30 s, 6000rpm). The coated film was then incubated in 2 mM of N-Ethyl-N-(3-(dimethylaminopropyl) carbodiimide (EDC; Fluka) and 5 mM of N-hydroxysuccinimide (NHS; Sigma Aldrich) (EDC/NHS) solution for 15 min, followed by 0.01 µM Ab immobilization. Figure 4a–c depicts the SPR signal for the Au, Au/Ab, and Au/CdSQDs-rGO/Ab sensor film generated by the detection of 10 pM DENV solution. As expected, no response was observed with the gold film without immobilization of Ab. From Figure 4b, the shift in SPR resonance angle can be observed when a gold film was immobilized with Ab. Subsequently, the shift in SPR resonance angle increased rapidly by 0.0438° due to the immobilization of the antibodies to the sensor surface, thus confirming the potential of Au/CdSQDs-rGO/Ab sensor film as a DENV-sensing material. 

**Figure 5:**
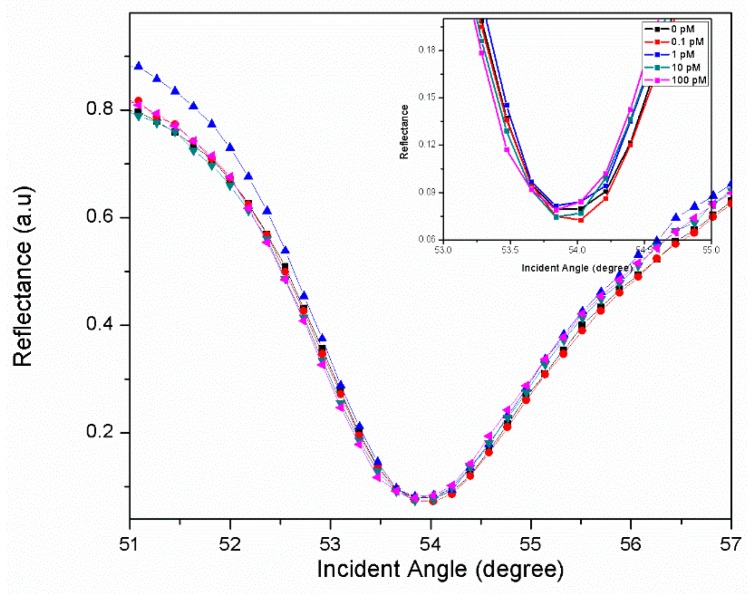
Optical reflectance for DENV concentrations (0.1–100 pM) in contact with Au/CdSQDs-rGO/Ab layer (inset: the zoomed in graph).

Further, the developed sensor film was exposed to various concentrations of DENV solution ranging from 0.1 pM to 100 pM, as shown in Figure 5. The DENV solutions were injected one after another into the flow chamber to be in contact with the sensor film for 20 min under room temperature. Prior to that, the phosphate buffer saline (PBS) solution was first injected into the flow chamber to obtain the reference SPR signal. The obtained resonance angle was 53.954°. When 0.1 pM DENV solution was subsequently introduced, the resonance angle was right-shifted to 53.968°. The increase in the resonance angle indicated that the DENV antigen had been bound onto sensor film through the specific interaction between DENV and monoclonal antibodies. This phenomenon can cause changes in sensor film thickness, and, hence, increases its refractive index. The changes in SPR angle were greater when the higher concentration of DENV solution (1 pM–100 pM) were introduced. However, the change in SPR angle resulted in a left-shift in resonance angle. It was apparent that the immobilization of biorecognition molecules was moving further apart from the previous binding, thus decreases the plasmonic coupling [87]. The limit of quantitation obtained with the proposed sensor was 0.1 pM DENV solution. 

**Figure 6:**
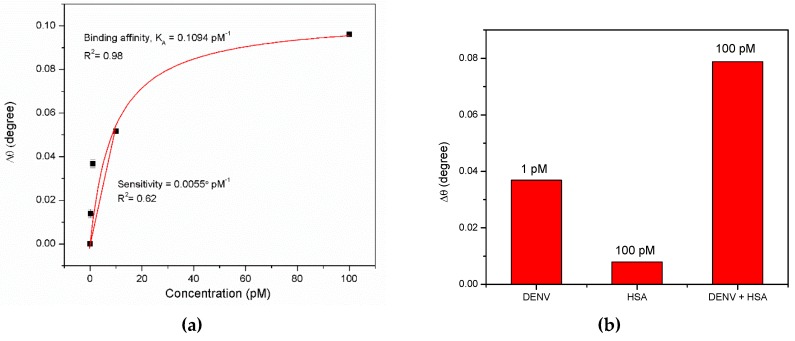
Relationship between the SPR angle shift (Δθ) and DENV concentration. (**a**) Langmuir and linear fitting; (**b**) Selectivity tests.

The reaction between the sensor film and the DENV antigen was evaluated using the Langmuir model, which can be expressed as follows:

(3) $$\Delta \theta =\frac{\Delta \theta_{\max}}{K_{D}+C},$$

where *Δθ*_max_ is the maximum SPR shift at the saturation, C is the concentration of DENV, and K_D_ is the equilibrium dissociation constant. As shown in Figure 6a, the fitting graph yielded a K_D_ value of 9.13 pM with R^2^ of 0.98. Moreover, the binding affinity, *K_A_*, of the sensor film to the DENV antigen, determined as the inverse proportional to *K_D_*, was 0.10948 pM^−1^. The sensitivity of the sensor film was then determined by plotting a linear graph from 0 pM to 10 pM DENV solution. The linear fit showed a good response with the sensitivity value of 0.0055°/pM (R^2^ = 0.62). The selectivity test was also tested by detecting 100 pM human serum albumin (HSA), and the mixture of HSA and DENV solution. As shown in Figure 6b, the resonance angle shift for DENV antigen was higher than that for HSA, which indicated a high selective binding towards the immobilized antibodies on the sensor film. Aside from that, as it can be seen, the resonance angle shift for the detection of the DENV + HSA solution was greatly increased due to the non-specific binding of the high molecular weight of HSA.

### 3.2. Graphene-SPR Based Materials for Metal Ion Detection

**Figure 7:**
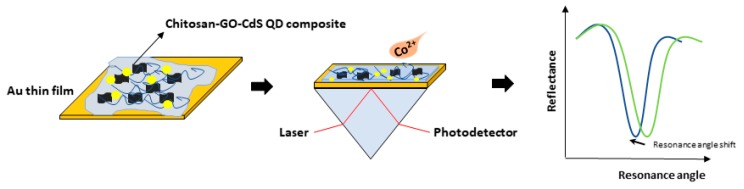
Schematic diagram of an optical biosensor for cobalt detection.

**Figure 8:**
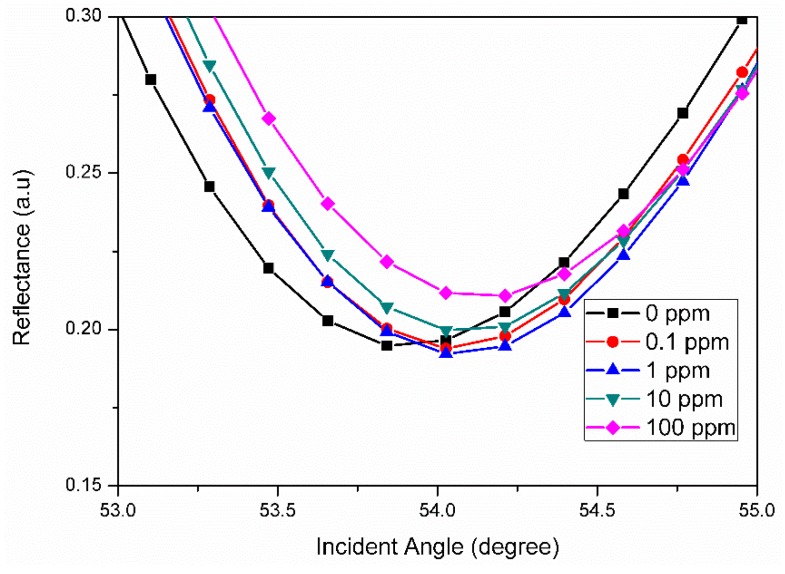
SPR optical curves for different concentration of cobalt ion (0, 0.1, 1, 10, and 100 ppm) in contact with the chitosan-GO-CdS QDs sensor layer.

The surface plasmon resonance technique integrated with graphene oxide-based material can also be applied for metal ion detection, as illustrated in Figure 7. Cobalt is a trace element that can be found widely in nature. One of the biological functions of cobalt is as a metal component of vitamin B12, also known as cyanocobalamin. However, excessive intake of cobalt and other cobalt compounds can be toxic to the human body, as reported by previous researchers [88,89,90]. Therefore, chitosan-graphene oxide-cadmium sulfide quantum dots (Chitosan-GO-CdS QDs) composite thin film was developed as a sensor layer for cobalt ion detection via surface the plasmon resonance sensor. The potential of the graphene oxide based sensor layer for biosensor application to detect cobalt ion was evaluated by exposing the sensor layer with deionized water and also with the different concentrations of cobalt ion (0.1 ppm, 1 ppm, 10 ppm, and 100 pm). Initially, deionized water was used to obtain the reference SPR signal, and then was furthered by introducing different concentration of cobalt ion on the sensor layer. The SPR curves of the experiment were obtained, as shown in Figure 8. From the SPR curves, the resonance angles of the deionized water, 0.1, 1, 10, and 100 ppm of cobalt ion obtained were 53.934°, 53.952°, 54. 025°, and 54.057°, respectively, as shown in Figure 7. The introduction of the cobalt ion on the sensor layer caused the resonance angle to shift to the right, and, as the concentration of cobalt ion was increased, the resonance angle shift also increased. This result confirmed the potential of the sensor layer to detect cobalt ion in low and higher concentrations.

**Figure 9:**
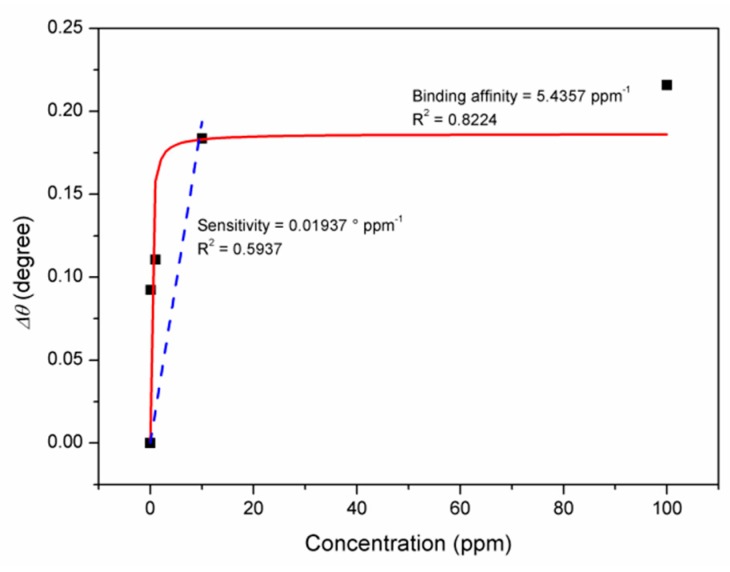
Data plotting the angle shift against the cobalt ion concentration fitted with Langmuir equation and linear fitting.

The angle shift value was then calculated and a graph was plotted for the degree of the resonance angle against concentration, as shown in Figure 9. The plotted data was then fitted with the Langmuir equation to obtain the binding affinity, *K_A_* and the maximum angle shift at the saturation of the SPR curves, *Δθ*_max_. From the Langmuir fitting, the sensor layer showed a high binding affinity toward the cobalt ion, with an affinity constant of 5.4357 ppm^−1^ with an R^2^ value of 0.8224. The high binding affinity of the sensor layer toward the cobalt ion may due to the electrostatic force between the graphene oxide sheets and also the CdS QD element. Complementary to this, the Langmuir fitting also showed that the saturation value of the sensor layer occurred when the concentration of the cobalt reached 10 ppm, with a maximum angle shift of 0.1865. In addition, the sensitivity of the sensor was 0.01937 ° ppm^−1^, which was determined by obtaining the slope of the linear fitting of the data from 0 to 10 ppm. These results show that the sensor layer has a great potential to detect cobalt ion.

## 4. Conclusions

In this study, a quantitative technique for the rapid and quantitative detection of DENV and Co^2+^ was developed using graphene-SPR-based materials. The graphene-based materials of Au/CdSQDs-rGO/Ab and Chitosan-GO-CdS QDs were able to differentiate the concentration of DENV and Co^2+^ from 0.1 to 100 pM and from 0.1 to 100 ppm, respectively. Besides that, the graphene-SPR-based sensor showed a good sensitivity of 0.0055° pM^−1^ and 0.0193° ppm^−1^ for DENV and Co^2+^, respectively. The reaction between the graphene-based sensor film with DENV and Co^2+^ were also evaluated using the Langmuir model. Interestingly, both the DENV and Co^2+^ have a good binding affinity towards the graphene-based film, i.e., 0.10948 pM^−1^ and 5.4537 ppm^−1^, respectively. On the other hand, the graphene-based material showed a higher sensitivity towards DENV compared to HSM. The DENV was also able to be differentiated in the mixture of HSM, and, hence, verified that the Au/CdSQDs-rGO/Ab had a good selectivity towards DENV. Therefore, the above results proved that the graphene-SPR-based sensor was successfully developed for health and environmental monitoring application in sensing DENV and Co^2+^.

## Figures and Tables

**Table 1:** Graphene- surface plasmon resonance (SPR)-based materials in biological applications.

Sensor Layer	Target	Limit of Detection	Sensitivity	Reference
Graphene	α-thrombin	0.05 nM	-	[68]
Au/SAM/Graphene/	Tuberculosis bacillus	-	-	[69]
Ag/Graphene-MoS2	ssDNA	-	-	[70]
Graphene	Mycobacterium tuberculosis (cssDNA)	28 fM	-	[71]
RGO	Rabbit IgG	0.0625 µg/mL	-	[72]
Au/GO-COOH	Anti BSA	0.01 pg/mL	-	[73]
Cr/Au/MoS2/Graphene	Urea	-	230°/RIU	[74]
Au/SAM/GO/3ABA	Galectin-3	2.0 ng/mL	-	[75]
Au/Graphene	Glucose	-	1227 nm/RIU	[76]
	DNA	0.1 nM	-	

**Table 2:** Graphene-SPR-based materials in metal ions sensing.

Active Layer	Metal Ions	Limit of Detection	Sensitivity	References
Au/CS/GO	Pb^2+^	0.03 ppm	1.11200° ppm^−1^	[77]
AuNPs/GO/PANI	Pb^2+^	0.03 ppm	-	[78]
Au-Ag-Au CS-GO	Pb^2+^	0.1 ppm	2.05° ppm^−1^	[79]
Au/Ag/Au/CS-GO	Hg^2+^	0.1 ppm	1.66° ppm^−1^	[80]
C-GO-V	K^+^	0.001 ppm	0.00948° ppm^−1^	[81]
CTA-NCC/GO	Cu^2+^	0.01 ppm	3.271° ppm^−1^	[82]
CTA-NCC/GO	Ni^2+^	0.01 ppm	1.509° ppm^−1^	[83]
Cs/CGQDs	Hg^2+^	0.5 ppm	0.00062° ppm^−1^	[84]
